# The association between brominated flame retardants and serum testosterone levels in American adult men: NHANES 2013–2016

**DOI:** 10.3389/fpubh.2025.1589047

**Published:** 2025-07-17

**Authors:** Xin Li, Mutong Chen, Qing Zheng, Zixuan Wang, Dini Lin, Mengmeng Peng

**Affiliations:** ^1^Department of Gastrointestinal Surgery, The First Affiliated Hospital of Shantou University Medical College, Shantou, China; ^2^Cancer Hospital of Shantou University Medical College, Shantou, China; ^3^Department of Endocrinology, The Third Affiliated Hospital of Wenzhou Medical University, Ruian, China; ^4^Wenzhou Key Laboratory for the Diagnosis and Prevention of Diabetic Complication, Wenzhou, China

**Keywords:** brominated flame retardants, serum testosterone, endocrine disrupting, NHANES, sex hormone binding globulin

## Abstract

**Background:**

Brominated flame retardants (BFRs), especially polybrominated diphenyl ethers (PBDEs), are commonly utilized, yet their possible endocrine-disrupting effects have sparked significant concerns. Nonetheless, the link between exposure to BFRs and serum testosterone levels in adult males is still not well comprehended.

**Methods:**

We analyzed data from 1,150 men aged ≥20 years from the National Health and Nutrition Examination Survey (NHANES) in 2013–2016. Serum concentrations of BFRs (PBDE congeners: PBDE-28, PBDE-47, etc.) and testosterone levels were measured via mass spectrometry and liquid chromatography–tandem mass spectrometry, respectively. Free testosterone (FT) and bioavailable testosterone (BAT) were calculated using the Vermeulen equation, based on measured total testosterone (TT), sex hormone-binding globulin (SHBG), and serum albumin concentrations. Linear regression models were used to evaluate the association between BFRs and TT, BAT, FT, and SHBG, adjusting for confounders including age, race, and lifestyle factors. We also evaluated potential associations modified by age, and conducted a sensitivity analysis to assess the robustness of the observed associations.

**Results:**

After all continuous variables were log2-transformed and potential confounders were adjusted, significant inverse associations were found between PBDE-28 and PBDE-47 levels with TT (*β* = −0.641, 95% CI: −1.098, −0.185) and FT (*β* = −0.883, 95% CI: −1.616, −0.149). Specifically, in the stratified analysis, older men (≥60 years) showed stronger associations between PBDE-28 and PBDE-47 exposure with lower testosterone levels (*β* = −0.892, 95% CI: −1.472, −0.311 for PBDE-28 and *β* = −0.695, 95% CI: −1.199, −0.191 for PBDE-47). Sensitivity analysis confirmed that PBDE-28 and PBDE-47 were consistently associated with reduced testosterone and free testosterone levels, with the associations remaining significant even after adjusting for potential co-exposures and lifestyle factors.

**Conclusion:**

Our findings suggest that exposure to PBDE-28 and PBDE-47 is associated with lower testosterone levels, particularly in older men. These results highlight the potential reproductive risks posed by BFR exposure, warranting further investigation into the long-term health impacts.

## 1 Introduction

Brominated flame retardants (BFRs) consist of various brominated organic substances that are incorporated into diverse materials to diminish the potential for fire hazards. These compounds can be classified into three primary categories according to how they bind with polymers: brominated monomers, reactive agents, and additives (1, 2). Due to the non-covalent nature of their binding to polymers, BFRs are known to continuously leach out from products and permeate into the surrounding environment (3). Numerous epidemiological studies have shown a significant link between exposure to brominated flame retardants (BFRs) and serious health issues or disturbances related to the nervous system, reproductive system, thyroid activity, and liver health (3–5). Polybrominated diphenyl ethers (PBDEs) are among the most widely used brominated flame retardants (BFRs) and are regarded as reactive BFRs, which are thought to pose a heightened risk to human health (1, 6). Commercial PBDE formulations are made up of a combination of congeners, which typically feature pentabromodiphenyl ethers (Penta-BDEs), octabromodiphenyl ethers (Octa-BDEs), and decabromodiphenyl ethers (Deca-BDEs). Penta-BDEs and Octa-BDEs were discontinued in the United States in 2004, while the production of Deca-BDEs ceased in 2013 (7). Although BFRs, including PBDEs, are being phased out, the persistence in consumer durables, food and indoor dust suggests that human exposure to these compounds will continue (8–12). As lipophilic molecules, BFRs can also accumulate in organisms, leading to biological amplification in the food chain (13). Moreover, the control of BFRs leading to the restoration of toxicokinetic dynamics does not necessarily guarantee the restoration of toxicodynamic dynamics, which highlights the fact that even limited early exposure might result in enduring consequences (14). It is foreseeable that human health will remain potentially affected by BFRs for decades (15).

Testosterone is the main male sex hormone, mostly secreted by the testes, with a small amount secreted by the adrenal glands (16). Normal levels of testosterone play an important role in male health, exerting significant effects on male sexual characteristics, brain functionality, muscle quality, and bone density (17, 18). Reduced serum testosterone (equal to or below 300 ng/dL) commonly manifests as decreased frequency of sexual thoughts and desire, increased body weight, and impaired erectile function (19, 20). Additionally, low testosterone levels is associated with diabetes, depressive symptoms, fatigue, as well as an increased risk of cardiovascular disease (21–26). In the United States, the prevalence of low testosterone levels among men aged 45 and above is as high as 40%, and the proportion is projected to increase in the coming decades (27). Studies have shown that many factors are associated with decreased testosterone levels, including advancing age, obesity, sedentary lifestyle, alcohol consumption, and the use of medications (28, 29). Moreover, research has also uncovered the possible influence of environmental endocrine-disrupting chemicals (EDCs) on the development of low testosterone, alongside these risk factors (30, 31). Given the significance of androgens in the general health of adult men, identifying factors that influence hormone levels has become a crucial issue in men’s well-being (32).

Research both *in vitro* and *in vivo* has been carried out to evaluate how BFRs affect testosterone levels. However, these studies mainly rely on laboratory data, and the findings remain perplexing without definitive conclusions. According to previous studies, the administration of BDE209 has been found to impact the production of testicular steroids and spermatogenesis in adult or prepubertal mice, resulting in a significant reduction in serum testosterone levels (33–35). A different investigation has demonstrated that mature male rats exposed to a complex mixture of three commercial BDE compounds—specifically DE-71 (52.1%), DE-79 (0.4%), and Deca-BDE-209 (44.2%)—along with hexabromocyclododecane (HBCDD at 3.3%), exhibited no notable impact on serum testosterone levels (36). Additionally, the limited number of epidemiological studies available is insufficient to fully elucidate the relationship between BFRs and testosterone, lacking generalizability to the adult men in the United States. In 2013, a study uncovered a positive correlation between the concentration of Octa-BDEs (the combined total of PBDE-183 and PBDE-201) in indoor dust and testosterone levels among males recruited from an infertility clinic in the Boston area. Conversely, there is an inverse relationship between the concentration of Deca-BDEs and testosterone levels (37). In a cross-sectional study conducted on fertile males from Greenland, Poland, or Ukraine, it was observed that environmental exposure to BDE-47 and BDE-153 was not associated with any alterations in testosterone levels (38). This study employed a significant representative sample from the United States to explore the association between BFR exposure and testosterone levels.

## 2 Materials and methods

### 2.1 Data sources

The data utilized in this study were sourced from the National Health and Nutrition Examination Survey (NHANES), a survey that holds national representativeness and is carried out by the National Center for Health Statistics (NCHS). NHANES makes use of a stratified, multistage probability sampling framework to evaluate the health and nutritional standing of the non-institutionalized civilian populace in the United States (39). For this analysis, data were restricted to 2013–2014 and 2015–2016 continuous data cycles, which included comprehensive information on BFRs and serum testosterone levels. To ensure reliable estimates, we combined the two survey cycles for our analyses. All data were gathered in accordance with the standardized procedures set by the NCHS. The study was approved by the NCHS Research Ethics Review Board, and every participant gave their written informed consent prior to getting involved.

### 2.2 Study population

**Figure 1:**
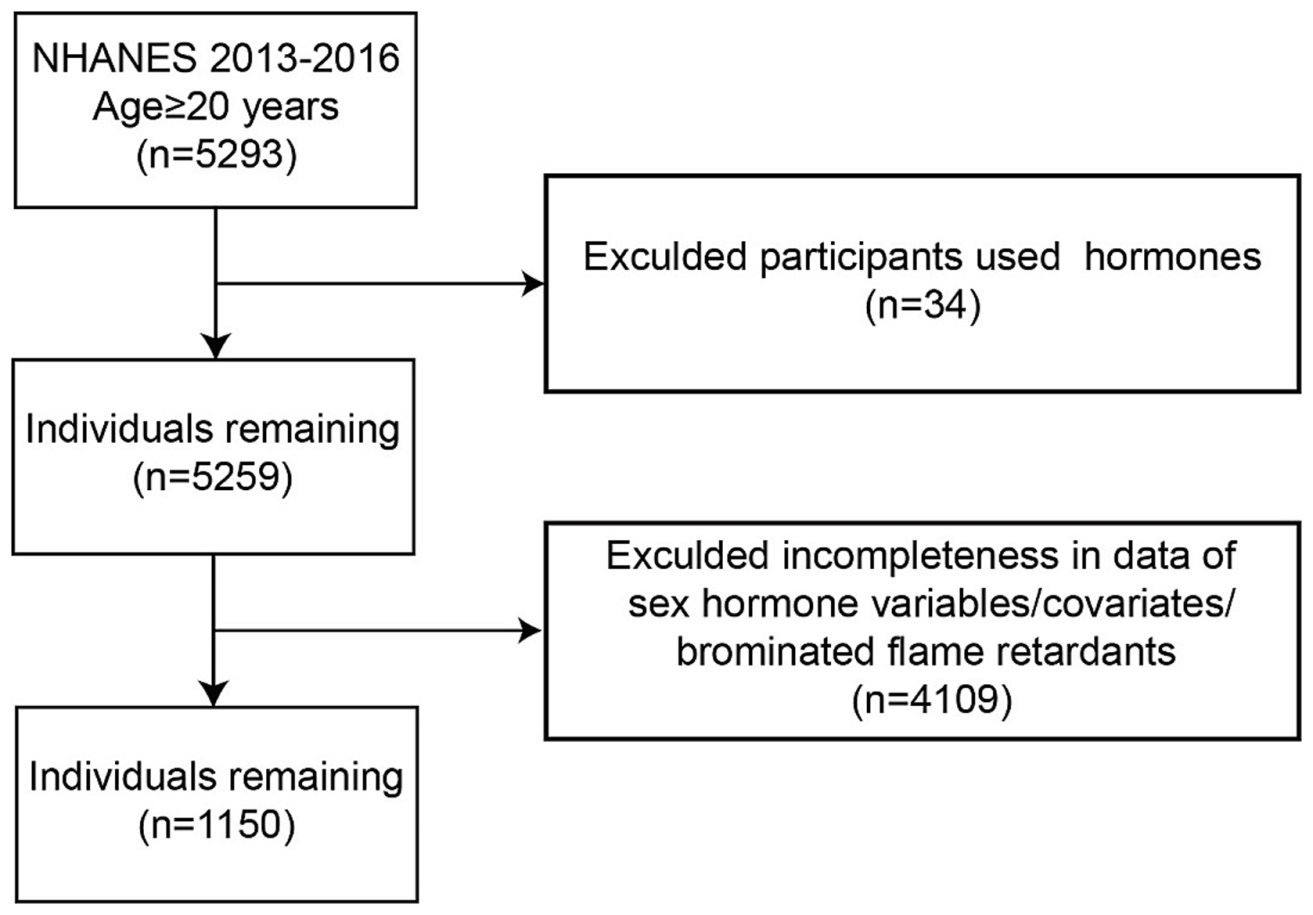
Flow diagram of the screening and enrollment of study participants.

Data were obtained from the 2013–2014 and 2015–2016 continuous NHANES cycles. The initial dataset included 5,293 adult men aged 20 years or older. Participants were excluded if they were using hormone therapy (*n* = 34) or had missing data on sex hormone variables, covariates, or serum BFRs. Following the implementation of these exclusion criteria, the final analysis comprised a total of 1,150 participants. Furthermore, PBDE congeners were selected based on a criterion of having greater than 50% detection rates in both the 2013–2014 and 2015–2016 NHANES cycles (Figure 1).

### 2.3 Measurement of serum BFRs and exposure to BFRs

BFRs concentrations were measured in NHANES using automated liquid–liquid extraction, followed by sample cleanup and analysis with isotope dilution gas chromatography coupled with high-resolution mass spectrometry (ID-GC/HRMS) (40). To ensure the stability of model, only BFR congeners with detection rates above 50% were included in the primary analysis. The selected BFRs included 2,4,4-Tribromodiphenyl ether (PBDE28) and 2,2,4,4-Tetrabromodiphenyl ether (PBDE47). All BFR concentrations underwent log transformation because of their skewed distribution to the right. The exposure levels were divided into quartiles, with the reference group in regression models being the lowest quartile.

### 2.4 Outcome measurement

In the NHANES study, the levels of serum total testosterone (TT) and estradiol (E2) were gaged. This was done through isotope dilution liquid chromatography and tandem mass spectrometry, with the measurements taken at a solitary time point, be it in the morning, afternoon, or evening. The levels of Sex hormone-binding globulin (SHBG) were determined through its interaction with immuno-antibodies. Subsequently, the products of this reaction were gaged by means of chemiluminescence detection, which utilized a photomultiplier tube. The free androgen index (FAI) was calculated as TT (ng/dL) divided by SHBG (nmol/L). Additionally, the TT/E2 ratio was used as an indirect marker of circulating free testosterone (FT) and aromatase activity. Furthermore, FT and bioavailable testosterone (BAT) were computed using the Vermeulen equation, which estimates FT and BAT based on measured TT, SHBG, and serum albumin concentrations. The Vermeulen methodology provides a reliable assessment of testosterone fractions that are available for biological activity (41). The primary outcome of this study was the association between BFRs exposure and serum testosterone levels, with testosterone deficiency (TD) defined as TT < 300 ng/dL, in accordance with the American Urological Association (AUA) guidelines.

### 2.5 Covariates

In the analysis, a number of categorical covariates were incorporated, taking into account their possible links with both BFR exposure and serum testosterone levels. Age was split into three brackets: 20 to 40 years old, 40 to 60 years old, and over 60 years old. Race and ethnicity were grouped as Mexican American, Non-Hispanic Black, Non-Hispanic White, Other Hispanic, and other races. Socioeconomic status was gaged using the poverty-income ratio (PIR). It was divided into low-income (≤1.3), middle-income (greater than 1.3 up to 3.5), and high-income (greater than 3.5). Education level was sorted into high school or below, some college experience, and college graduate or higher. Marital status was classified as Married/Living with a Partner, Never Married, and Widowed/Divorced/Separated. Covariates related to health included hypertension, which was defined as self-reported use of antihypertensive medications or measured systolic blood pressure of at least 140 mmHg or diastolic blood pressure of at least 90 mmHg. Diabetes was defined as self-reported diabetes or a fasting plasma glucose level of at least 126 mg/dL or glycated hemoglobin (HbA1c) of at least 6.5%. Lifestyle factors covered leisure-time physical activity (LTPA), which was put into three categories. There were the inactive (those who did not take part in any leisure-time physical activity), the insufficiently active (people who engaged in moderate activity one to five times a week with metabolic equivalents [METs] between 3 and 6, or vigorous activity one to three times a week with METs over 6), and the active (those who met and exceeded the above criteria) (42). Smoking status was categorized into two groups: those who had never smoked and those who were either current or former smokers. Alcohol consumption, on the other hand, was ascertained via self-reporting. People who drank a minimum of 12 standard alcoholic beverages in any particular year were labeled as alcohol drinkers. These variables were then included in regression models to account for possible confounding factors that could influence the relationship between BFR exposure and serum testosterone levels.

### 2.6 Statistical analyses

In the descriptive analyses, we calculated median (interquartile range, IQR) ± standard deviation (SD) for continuous variables and the frequencies for categorical variables. To investigate the association between BFR exposure and sex hormone levels, we employed crude model and adjusted model, adjusting for potential confounders including age, race/ethnicity, poverty-income ratio, education, marital status, smoking status, alcohol drinking, hypertension, diabetes, and leisure-time physical activity. Furthermore, we employed GVIF to detect multicollinearity in the regression analysis by assessing the variance inflation of each independent variable, which helps evaluate the reliability and stability of the model. All covariates included in the models had GVIF values below 5.0, indicating acceptable levels of multicollinearity. Due to the right-skewed distribution of continuous variables, including BFRs and sex hormone levels, all continuous variables were log2-transformed prior to analysis. Only BFRs with a detection rate >50% were included in the analysis. Although age was categorized into three groups for descriptive purposes, a binary stratification (<60 years and ≥60 years) was used in effect modification analysis based on literature indicating accelerated age-related hormonal changes after 60 years of age (43).

Additionally, Sensitivity analysis was conducted to assess the robustness of the observed associations between BFRs and hormone levels, with a particular focus on testosterone and SHBG. We used lipid adjusted concentrations as a replacement for the original measured concentration of BFRs to reduce variability since differences in individuals’ serum lipid concentrations are canceled out (44, 45). For BFRs that were detectable in less than 50% of the samples, multiple regression analyses were conducted by categorizing exposure into detectable (> LOD) and nondetectable (< LOD) levels.

All statistical analyses were conducted using Empower®.^1^ A two-tailed *p*-value <0.05 was considered statistically significant.

## 3 Results

### 3.1 Participant characteristics

A total of 1,150 participants were included in the analysis (Table 1). The median age was evenly distributed across the three groups: 35.13% were aged 20–40 years, 31.13% were 40–60 years, and 33.74% were 60 years or older. The majority were Non-Hispanic White (39.57%), followed by Non-Hispanic Black (20.78%), Mexican American (14.96%), and other racial/ethnic groups (24.70%).

**Table 1:** Population characteristics of American adult men in NHANES 2013–2016 (*n* = 1,150).

Participant characteristics		Median (IQR) or Mean ± SD
Age (year)	[20, 40]	404 (35.13)
	[40, 60]	358 (31.13)
	[60]	388 (33.74)
Race ethnicity old (%)	Mexican American	172 (14.96)
	Non-Hispanic Black	239 (20.78)
	Non-Hispanic White	455 (39.57)
	Other Hispanic	122 (10.61)
	Others	162 (14.09)
Marital status all cycle (%)	Married/Living with Partner	760 (66.09)
	Never married	219 (19.04)
	Widowed/Divorced/Separated	171 (14.87)
Poverty income ratio breaks (%)	<1.4	353 (30.70)
	1.4–3.5	418 (36.35)
	>3.5	379 (32.96)
Education (%)	High school or less	524 (45.57)
	Some college	332 (28.87)
	Colleage graduate or higher	294 (25.57)
Time of venipuncture (%)	Afternoon	400 (34.78)
	Evening	173 (15.04)
	Morning	577 (50.17)
BMI type (%)	<25	306 (26.61)
	25–29.9	436 (37.91)
	>29.9	408 (35.48)
Smoke (%)	No	552 (48.00)
	Yes	598 (52.00)
Alcohol user (%)	No	109 (9.48)
	Yes	1,041 (90.52)
LTPA BINDED (%)	No	547 (47.57)
	Moderate	290 (25.22)
	Vigorous	313 (27.22)
Hypertension (%)	No	635 (55.22)
	Yes	515 (44.78)
Diabetes mellitus (%)	No	903 (78.52)
	Yes	247 (21.48)
Brominated flame retardants	PBDE17 (mean (SD))	3.85 (1.68)
	PBDE28 (mean (SD))	3.88 (0.72)
	PBDE209 (mean (SD))	2.46 (0.87)
	PBDE47 (mean (SD))	6.61 (0.88)
	PBDE85 (mean (SD))	1.08 (0.92)
	PBDE99 (mean (SD))	4.27 (1.01)
	PBDE100 (mean (SD))	4.36 (0.85)
	PBDE153 (mean (SD))	6.00 (0.92)
	PBDE154 (mean (SD))	0.86 (0.90)
Sex hormones	Total testosterone (nmol/L) (mean (SD))	14.58 (6.38)
	Free testosterone (nmol/L) (mean (SD))	0.26 (0.11)
	Cbat (nmol/L) (mean (SD))	6.00 (2.63)
	Sex hormone binding globulin (nmol/L) (mean (SD))	45.21 (25.00)
	Estradiol (pg/ml) (mean (SD))	25.32 (9.96)

IQR, Inter quartile range. Poverty-Income Ratio (PIR): A measure of socioeconomic status, divided as follows: <1.4: Low-income group; 0.4–3.5: Middle-income group; >3.5: High-income group. Alcohol User: Defined as consuming at least 12 standard alcoholic beverages in a year. Hypertension: Defined as the self-reported use of antihypertensive medications or a measured systolic blood pressure ≥140 mmHg or diastolic blood pressure ≥90 mmHg. PBDE17–2,2′,4,4′,5,5′-hexabromobiphenyl (pg/g); PBDE28–2,4,4′-tribromodiphenyl ether (pg/g); PBDE47–2,2′,4,4′-tetrabromodiphenyl ethr (pg/g); PBDE85–2,2′,3,4,4′-pentbromodiphenyl ethr (pg/g); PBDE99–2,2′,4,4′,5-pentabromodiphnyl ethr (pg/g); PBDE100–2,2′,4,4′,6-pentabromodiphyl ether (pg/g); PBDE153–2,2′,4,4′,5,5′-hxbromodiphnyl ethr (pg/g); PBDE154–2,2′,4,4′,5,6′-hxabromodiphyl ethr (pg/g); PBDE209, Decabromodiphenyl ether (pg/g). Total testosterone (nmol/L); Free testosterone (nmol/L); Cbat (nmol/L); Sex hormone binding globulin (nmol/L); Estradiol (pg/ml).

Regarding socioeconomic factors, 66.09% were married or living with a partner, while 30.70% had a poverty-income ratio below 1.4. Educational attainment varied, with 45.57% having a high school education or less and 25.57% holding a college degree or higher. Among health-related characteristics, 35.48% had a BMI ≥ 30, 44.78% had hypertension, and 21.48% had diabetes. Additionally, 52.00% were current or former smokers, and 90.52% reported alcohol consumption.

Log2-transformed PBDE levels showed high variability, with mean (SD) values of 6.61 (0.88) for PBDE47, 6.00 (0.92) for PBDE153, and 4.36 (0.85) for PBDE100. Notably, PBDE concentrations were originally measured in pg/g lipid and as we mentioned before, they were log2-transformed to address skewed distributions. As a result, the transformed values are unitless but retain interpretive relevance in terms of fold-change. The mean (SD) testosterone level was 14.58 (6.38) nmol/L, while E2 was 25.32 (9.96) pg/mL.

### 3.2 Associations between brominated flame retardants and sex hormones

Table 2 presents the associations between PBDEs and sex hormone levels in American adult men. Regression coefficients for PBDEs and testosterone levels are presented based on log2-transformed data, reflecting the effect size per doubling of the BFR concentration. After adjusting for potential confounders, we observed that PBDE-28 and PBDE-47 were significantly associated with lower levels of TT, FT, and Cbat (*p < 0.05*).

**Table 2:** Associations between brominated flame retardants and sex hormones in American adult men in NHANES 2013–2016 [Coefficients (95% confidence interval)] (*n* = 1,150).

Brominated flame retardants^a^	Total testosterone^b^	Free testosterone	Cbat	Sex hormone binding globulin
PBDE17	−0.033 (−0.317, 0.250)	−0.160 (−0.615, 0.295)	−0.037 (−0.144, 0.069)	0.225 (−0.791, 1.240)
PBDE28	−0.641 (−1.098, −0.185) *	−0.883 (−1.616, −0.149) *	−0.207 (−0.379, −0.035) *	−1.332 (−2.970, 0.305)
PBDE47	−0.468 (−0.887, −0.049) *	−0.800 (−1.473, −0.127) *	−0.188 (−0.345, −0.030) *	−0.687 (−2.190, 0.817)
PBDE85	−0.220 (−0.608, 0.167)	−0.466 (−1.088, 0.155)	−0.109 (−0.255, 0.036)	−0.694 (−2.081, 0.693)
PBDE99	−0.272 (−0.634, 0.090)	−0.494 (−1.075, 0.087)	−0.116 (−0.252, 0.020)	−0.594 (−1.891, 0.704)
PBDE100	−0.314 (−0.744, 0.117)	−0.454 (−1.144, 0.237)	−0.106 (−0.268, 0.056)	−1.005 (−2.546, 0.535)
PBDE153	−0.044 (−0.453, 0.365)	−0.170 (−0.826, 0.486)	−0.040 (−0.194, 0.114)	−0.113 (−1.577, 1.350)
PBDE154	−0.136 (−0.536, 0.263)	−0.457 (−1.097, 0.184)	−0.107 (−0.257, 0.043)	−0.247 (−1.678, 1.184)
PBDE209	−0.002 (−0.493, 0.490)	0.515 (−0.274, 1.303)	0.121 (−0.064, 0.305)	−1.214 (−2.973, 0.545)

a PBDE17–2,2′,4,4′,5,5′-hexabromobiphenyl (pg/g); PBDE28–2,4,4′-tribromodiphenyl ether (pg/g); PBDE47–2,2′,4,4′-tetrabromodiphenyl ethr (pg/g); PBDE85–2,2′,3,4,4′-pentbromodiphenyl ethr (pg/g); PBDE99–2,2′,4,4′,5-pentabromodiphnyl ethr (pg/g); PBDE100–2,2′,4,4′,6-pentabromodiphyl ether (pg/g); PBDE153–2,2′,4,4′,5,5′-hxbromodiphnyl ethr (pg/g); PBDE154–2,2′,4,4′,5,6′-hxabromodiphyl ethr (pg/g); PBDE209, Decabromodiphenyl ether (pg/g).

b Total testosterone (nmol/L); Free testosterone (nmol/L); Cbat (nmol/L); Sex hormone binding globulin (nmol/L); Estradiol (pg/ml).

*p < 0.05. Brominated flame retardants were log2-transformed. Estimates were adjusted for age (continuous), race/ethnicity (categorical), marital status (categorical), poverty income ratio (continuous), education level (categorical), time of venipuncture (categorical), BMI (categorical), smoking (categorical), drinking (categorical), ltpa binded (categorical), hypertension (categorical), diabetes mellitus (categorical).

Specifically, PBDE-28 showed a negative association with TT (*β* = −0.641, 95% CI: −1.098, −0.185), FT (*β* = −0.883, 95% CI: −1.616, −0.149), and Cbat (*β* = −0.207, 95% CI: −0.379, −0.035). Similarly, PBDE-47 was inversely associated with total testosterone (*β* = −0.468, 95% CI: −0.887, −0.049), FT (*β* = −0.800, 95% CI: −1.473, −0.127), and Cbat (*β* = −0.188, 95% CI: −0.345, −0.030). These findings suggest that higher PBDE-28 and PBDE-47 exposure may be linked to decreased androgen levels.

For other PBDE congeners, the associations with sex hormones were generally not statistically significant. Although negative trends were observed for PBDE-85, PBDE-99, PBDE-100, PBDE-153, and PBDE-154, their confidence intervals included zero, indicating no strong evidence of association. Additionally, PBDE-209 showed a weak positive association with FT (*β* = 0.515, 95% CI: −0.274, 1.303) but was not statistically significant. Overall, our results highlight PBDE-28 and PBDE-47 as the primary PBDEs associated with lower testosterone levels in adult men.

### 3.3 Stratified analysis

To explore potential effect modification by age, we conducted a stratified analysis for men aged <60 years and ≥60 years, with the results summarized in Table 3. Among men <60 years, no significant associations were observed between PBDEs and testosterone, FT, or CBAT levels. In contrast, among men ≥60 years, PBDE-28 (*β* = −0.892, 95% CI: −1.472, −0.311, P interaction = 0.0788) and PBDE-47 (*β* = −0.695, 95% CI: −1.199, −0.191, P interaction = 0.0627) were inversely associated with testosterone, while PBDE-100 also showed a negative association (*β* = −0.519, 95% CI: −1.032, −0.005), though the interaction was not statistically significant (P interaction = 0.1103). Similarly, FT levels were significantly lower in association with PBDE-17, PBDE-28, and PBDE-47 exposure among older men, with PBDE-28 exhibiting the strongest inverse relationship (*β* = −1.829, 95% CI: −2.783, −0.875, P interaction = 0.032). For CBAT, no notable associations were detected in younger men, whereas PBDE-17, PBDE-28, and PBDE-47 showed inverse associations in older men, with PBDE-28 (*β* = −0.429, 95% CI: −0.652, −0.205, P interaction = 0.032) and PBDE-47 (*β* = −0.291, 95% CI: −0.485, −0.096, P interaction = 0.0955) demonstrating the most pronounced effects. In terms of SHBG, a significant positive association was observed for PBDE-17 in older men (*β* = 1.773, 95% CI: 0.535, 3.010, P interaction = 0.1945), while no other PBDEs showed notable associations in either age group. Interaction analyses revealed that PBDE-28, PBDE-47, and PBDE-17 exhibited significant age-related differences in their effects, with inverse associations between PBDE exposure and testosterone, FT, and CBAT being more pronounced in men ≥60 years (P interaction < 0.1), suggesting that older adults may be more vulnerable to PBDE-related endocrine disruption.

**Table 3:** Associations between BFRs and sex hormones by age groups in American adult men in NHANES 2013–2016.

Brominated flame retardants	Age (year)	Testosterone	Free Testosterone	Cbat	Sex hormone binding globulin	Estradiol
PBDE17	<60	0.138 (−0.383, 0.659)	0.172 (−0.683, 1.027)	0.040 (−0.160, 0.241)	0.272 (−1.679, 2.223)	0.670 (−0.198, 1.538)
	≥60	−0.186 (−0.516, 0.145)	−0.873 (−1.416, −0.331)	−0.205 (−0.332, −0.078)	1.773 (0.535, 3.010)	−0.191 (−0.742, 0.360)
P for interaction		0.2953	**0.0392** ^*^	**0.0392** ^*^	0.1945	0.0945
PBDE28	<60	−0.066 (−0.803, 0.671)	−0.173 (−1.384, 1.038)	−0.041 (−0.324, 0.243)	−0.489 (−3.268, 2.290)	−0.452 (−1.685, 0.781)
	≥60	−0.892 (−1.472, −0.311)	−1.829 (−2.783, −0.875)	−0.429 (−0.652, −0.205)	0.322 (−1.866, 2.510)	−0.573 (−1.544, 0.398)
P for interaction		0.0788	**0.032** ^*^	**0.032** ^*^	0.6467	0.8774
PBDE47	<60	0.156 (−0.605, 0.917)	0.013 (−1.239, 1.266)	0.003 (−0.290, 0.297)	0.131 (−2.735, 2.998)	−0.561 (−1.834, 0.711)
	≥60	−0.695 (−1.199, −0.191)	−1.241 (−2.071, −0.411)	−0.291 (−0.485, −0.096)	−0.524 (−2.423, 1.376)	−0.229 (−1.072, 0.614)
P for interaction		0.0627	0.0955	0.0955	0.7035	0.6634
PBDE85	<60	−0.009 (−0.764, 0.746)	−0.246 (−1.490, 0.997)	−0.058 (−0.349, 0.234)	−0.154 (−2.990, 2.682)	−0.555 (−1.815, 0.704)
	≥60	−0.310 (−0.762, 0.142)	−0.557 (−1.301, 0.187)	−0.130 (−0.305, 0.044)	−0.972 (−2.669, 0.726)	0.257 (−0.497, 1.011)
P for interaction		0.4948	0.6685	0.6685	0.6209	0.2688
PBDE99	<60	−0.035 (−0.703, 0.633)	−0.231 (−1.330, 0.869)	−0.054 (−0.312, 0.204)	−0.186 (−2.696, 2.324)	−0.538 (−1.652, 0.576)
	≥60	−0.371 (−0.805, 0.062)	−0.661 (−1.374, 0.052)	−0.155 (−0.322, 0.012)	−0.603 (−2.232, 1.025)	0.167 (−0.556, 0.890)
P for interaction		0.398	0.5118	0.5118	0.7805	0.2887
PBDE100	<60	0.244 (−0.560, 1.048)	0.246 (−1.080, 1.573)	0.058 (−0.253, 0.369)	0.044 (−2.977, 3.066)	−0.673 (−2.016, 0.670)
	≥60	−0.519 (−1.032, −0.005)	−0.626 (−1.473, 0.220)	−0.147 (−0.345, 0.052)	−1.808 (−3.737, 0.120)	0.166 (−0.691, 1.024)
P for interaction		0.1103	0.2676	0.2676	0.3017	0.2926
PBDE153	<60	0.129 (−0.700, 0.958)	0.425 (−0.940, 1.789)	0.100 (−0.220, 0.419)	−0.585 (−3.692, 2.521)	−0.350 (−1.731, 1.032)
	≥60	−0.093 (−0.561, 0.374)	0.261 (−0.509, 1.031)	0.061 (−0.119, 0.242)	−1.729 (−3.482, 0.024)	0.388 (−0.391, 1.168)
P for interaction		0.6401	0.8346	0.8346	0.5218	0.3525
PBDE154	<60	0.237 (−0.518, 0.993)	−0.005 (−1.249, 1.239)	−0.001 (−0.293, 0.290)	0.474 (−2.363, 3.312)	−0.630 (−1.890, 0.629)
	≥60	−0.261 (−0.733, 0.211)	−0.569 (−1.346, 0.208)	−0.133 (−0.315, 0.049)	−0.755 (−2.527, 1.017)	0.186 (−0.601, 0.973)
P for interaction		0.2637	0.4428	0.4428	0.4627	0.2721
PBDE209	<60	−0.062 (−0.920, 0.796)	0.728 (−0.683, 2.138)	0.170 (−0.160, 0.501)	−1.335 (−4.544, 1.875)	1.479 (0.051, 2.908)
	≥60	0.064 (−0.556, 0.684)	1.006 (−0.013, 2.025)	0.236 (−0.003, 0.475)	−3.314 (−5.633, −0.994)	0.161 (−0.871, 1.193)
P for interaction		0.8111	0.749	0.749	0.3181	0.135

Brominated flame retardants were log2-transformed. Estimates were adjusted for age (continuous), race/ethnicity (categorical), marital status (categorical), poverty income ratio (continuous), education level (categorical), time of venipuncture (categorical), BMI (categorical), smoking (categorical), drinking (categorical), ltpa binded (categorical), hypertension (categorical), diabetes mellitus (categorical). *p < 0.05. The *p*-values that reached statistical significance are bolded.

Each model included one log2-transformed PBDE congener as the independent variable and serum hormone (total testosterone, free testosterone, or SHBG) as the dependent variable. Multiple linear regression models were used and all models were adjusted for covariates described in the Methods.

### 3.4 Sensitivity analysis

After the replacement of the original serum BFR concentration with a lipid-adjusted concentration, the result demonstrated that PBDE209 exposure was significantly associated with a reduction in TT (*β* = −0.566, 95% CI: −1.074, −0.059, *p* < 0.05), whereas PBDE47 exhibited a significant negative correlation with both FT (*β* = −0.037, 95% CI: −0.073, −0.001, *p* < 0.05) and Cbat (*β* = −0.009, 95% CI: −0.017, −0.000, *p* < 0.05). Although other BFRs showed predominantly negative estimates, their confidence intervals encompassed the null, suggesting weaker or non-significant associations. The wide confidence intervals observed for PBDE85 and PBDE154 indicate potential instability in the effect estimates, likely due to sample size limitations or individual variability. Sensitivity analysis was conducted using log2-transformed PBDE concentrations to ensure the robustness of the observed associations with testosterone levels. After accounting for key covariates like age, BMI, smoking status, and possible environmental co-exposures, these findings stayed consistent (Table 4).

**Table 4:** Associations between brominated flame retardants and sex hormones in American adult men by adjusted regression model.

Brominated flame retardants^a^	Total testosterone^b^	Free testosterone	Cbat	Sex hormone binding globulin
PBDE17	−0.017 (−0.039, 0.004)	−0.028 (−0.063, 0.008)	−0.006 (−0.015, 0.002)	−0.017 (−0.096, 0.061)
PBDE28	0.028 (−0.140, 0.196)	0.141 (−0.129, 0.411)	0.033 (−0.030, 0.096)	−0.163 (−0.765, 0.439)
PBDE47	−0.021 (−0.043, 0.002)	−0.037 (−0.073, −0.001)*	−0.009 (−0.017, −0.000)*	−0.039 (−0.119, 0.041)
PBDE85	−0.560 (−1.329, 0.209)	−0.948 (−2.182, 0.286)	−0.222 (−0.511, 0.067)	−1.728 (−4.481, 1.024)
PBDE99	−0.058 (−0.138, 0.021)	−0.110 (−0.237, 0.018)	−0.026 (−0.056, 0.004)	−0.129 (−0.414, 0.157)
PBDE100	−0.063 (−0.164, 0.038)	−0.092 (−0.253, 0.070)	−0.021 (−0.059, 0.016)	−0.266 (−0.627, 0.094)
PBDE153	0.011 (−0.023, 0.044)	0.013 (−0.042, 0.067)	0.003 (−0.010, 0.016)	−0.002 (−0.123, 0.119)
PBDE154	−0.558 (−1.568, 0.451)	−1.216 (−2.835, 0.404)	−0.285 (−0.664, 0.095)	−1.532 (−5.147, 2.082)
PBDE209	−0.566 (−1.074, −0.059)*	−0.778 (−1.593, 0.038)	−0.182 (−0.373, 0.009)	−1.606 (−3.425, 0.214)

Brominated flame retardants were log2-transformed. Estimates were adjusted for age (continuous), race/ethnicity (categorical), marital status (categorical), poverty income ratio (continuous), education level (categorical), time of venipuncture (categorical), BMI (categorical), smoking (categorical), drinking (categorical), ltpa binded (categorical), hypertension (categorical), diabetes mellitus (categorical). *p < 0.05.

aPBDE17–2,2′,4,4′,5,5′-hexabromobiphenyl (pg/g); PBDE28–2,4,4′-tribromodiphenyl ether (pg/g); PBDE47–2,2′,4,4′-tetrabromodiphenyl ethr (pg/g); PBDE85–2,2′,3,4,4′-pentbromodiphenyl ethr (pg/g); PBDE99–2,2′,4,4′,5-pentabromodiphnyl ethr (pg/g); PBDE100–2,2′,4,4′,6-pentabromodiphyl ether (pg/g); PBDE153–2,2′,4,4′,5,5′-hxbromodiphnyl ethr (pg/g); PBDE154–2,2′,4,4′,5,6′-hxabromodiphyl ethr (pg/g); PBDE209, Decabromodiphenyl ether (pg/g).

bTotal testosterone (nmol/L); Free testosterone (nmol/L); Cbat (nmol/L); Sex hormone binding globulin (nmol/L); Estradiol (pg/ml).

## 4 Discussion

Using a large and representative sample of American adult men, we observed a negative correlation between serum levels of BFRs and TT and FT levels. Importantly, our study found no association between BFRs and TD.

Our study revealed a significant relationship between PBDE-28 and PBDE-47 levels and decreased testosterone levels in adult men. Research to date has primarily focused on BDE-47, which is the most prevalent PBDE congener found in human tissues (46). The primary histopathological changes in the testes of BDE-47-treated animals include degeneration and necrosis of the seminiferous epithelium, shedding of necrotic spermatocytes and supporting cells, and collapsed necrotic tubules (47). Several mechanisms have been proposed for BDE-47-induced testicular damage, including induction of apoptosis, increased reactive oxygen species (ROS), and disruption of hormonal homeostasis (48–51). Specifically, exposure to BDE-47 strongly inhibited glutathione-associated enzymes (GPx, GST, and GSH), which may lead to increased peroxidation (52). BDE-47 downregulated multiple genes involved in steroid hormone synthesis in Leydig cells, such as 17βHSD, Hsd3b6, Star, Asah1, Dhcr24, and Cyb5r3, suggesting that BDE-47 may impair spermatogenesis by inhibiting testosterone production (46–48, 53). The analysis of single-cell RNA sequencing (scRNA-seq) data indicated a notable downregulation of Ncor1 and Kdm3a, implying that testicular injury induced by BDE-47 might occur via the alteration of androgen receptor signaling within supporting cells. Furthermore, BDE-47 significantly reduced the expression of key genes involved in cholesterol biosynthesis, such as DHCR24 and CYBR3. These findings further support the notion that BDE-47 inhibits testosterone synthesis, as cholesterol is the precursor for all steroid hormones (31). Additionally, BDE-47 interferes with thyroid homeostasis and disrupts testicular steroidogenesis (31, 54–56). In contrast to BDE-47, there is limited experimental evidence regarding BDE-28. However, some studies suggest that BDE-28 may bind tightly to the thyroid hormone receptor (TRα), disrupting thyroid hormone signaling and subsequently affecting normal sex hormone levels (56, 57).

In our study, people aged 60 or above showed a more pronounced decrease in testosterone levels after BFR exposure. A reasonable explanation is that with age, testosterone levels in older individuals are more susceptible to inflammatory environments, and their antioxidant defenses are diminished, making them more vulnerable to BFR effects. Inflammation is a hallmark of aging, and it has been observed in various organs (58, 59). Animal studies have shown that aging mice exhibit increased pro-inflammatory cytokines and overactive macrophages in the testes (60). This inflammatory microenvironment is not conducive to testosterone production, but anti-inflammatory treatments can enhance testosterone levels (61). Detecting pro-inflammatory cytokines within the aging human testis may lead to the creation of anti-inflammatory treatments aimed at alleviating the decline in fertility associated with aging. Additionally, oxidative stress is thought to play a role in the harmful mechanisms linked to PBDEs (62, 63). Mitochondria are recognized for their essential function in the control of oxidative stress. Dysfunctional mitochondria are a key characteristic of the aging process and contribute to the increase in oxidative stress (64). Aged mitochondria, with reduced antioxidant capacity, are unable to cope with ROS induced by PBDEs, exacerbating testicular damage and leading to a more significant decline in testosterone levels.

The vast majority of published work was based on experimental analysis *in vitro* or animal model. Limited human research has investigated the relationships between exposure to BFRs and levels of reproductive hormones. Certain studies have identified an inverse relationship between PBDEs and testosterone levels in males (37). A typical example is the findings of Makey et al. (2016), who also reported inverse associations between certain PBDE congeners, particularly BDE-153, and serum testosterone concentrations in North American men. Although their study was based on a smaller and more geographically limited population, the results provide supporting evidence for the endocrine-disrupting potential of PBDEs on male reproductive hormones (65). However, some studies did not observe significant associations (66). Furthermore, exposure data for newer BFRs (such as DBDPE and TBC) are still limited, and their long-term health effects are unclear (66–68). Related epidemiological evidence comes from small samples or specific exposed populations, leading to inconsistencies in the results. This is mainly due to the extensive diversity of BFR compounds, which exhibit significant variations in their metabolic rates, as well as the inherent challenges in precisely quantifying human exposure pathways. Furthermore, investigating hormone-related effects necessitates longitudinal monitoring through repeated blood or urine sample collection, a process that is often constrained by the scarcity of long-term tracking data.

Our study, utilizing real-world population data, has unveiled the potential health risks associated with chronic low-dose PBDE exposure in the general population. We identified a significant correlation between PBDE-28 and PBDE-47 concentrations and testosterone level reduction in adult males, with a particularly pronounced effect observed among older males. While previous research has predominantly focused on higher brominated congeners or other BFRs, our findings demonstrate the equally concerning endocrine-disrupting potential of lower brominated PBDEs. Furthermore, considering that older males naturally experience age-related testosterone decline, we hypothesize that PBDE exposure may exacerbate this process, potentially explaining the non-genetic factors contributing to the deterioration of reproductive health in this population.

This study holds significant public health implications. Based on our findings, preventive measures in household environments should focus on minimizing exposure to dust released from aging PBDE-containing furniture and electronic devices. We recommend comprehensive screening combining hormone levels assessment and pollutant load analysis for older males exhibiting both low testosterone levels and high PBDE exposure, enabling early identification of at-risk individuals and timely intervention strategies. Regrettably, current research predominantly emphasizes the neurotoxicity and carcinogenicity of BFRs, while the chronic low-dose effects on the reproductive system remain inadequately addressed, possibly due to public health prioritization.

This study acknowledges several limitations. Since our research relied on cross-sectional data derived from NHANES, it could only illustrate simultaneous relationships between PBDE exposure and testosterone levels, without confirming the temporal sequence of exposure and associated hormonal changes. Given the long half-life of PBDEs, concentration measurements at a single time point may not fully reflect long-term cumulative exposure, potentially underestimating the association strength between chronic exposure and reproductive impairment. Since PBDE exposure levels are closely related to lifestyle factors, the generalizability of our findings to non-US populations remains unverified. Furthermore, while our study focused exclusively on PBDEs, with traditional BFRs being phased out, future research should investigate the effects of emerging brominated or organophosphate flame retardants.

## 5 Conclusion

This study establishes a significant link between BFRs and hormone levels in American adult males. We have demonstrated that exposure to PBDE-28 and PBDE-47 is associated with decreased testosterone levels, particularly evident in the older male population. These findings highlight the endocrine-disrupting potential of PBDE congeners and emphasize the necessity for further investigation into their long-term health implications.

## Data Availability

The datasets presented in this study can be found in online repositories. The names of the repository/repositories and accession number(s) can be found in the article/supplementary material.

## Footnotes

^1^ www.empowerstats.com
